# Supplementary material for: Epigenetic Profiling in the Saliva of Obese Pregnant Women
Source: Nutrients. 2022 May 19;14(10):2122. doi: 10.3390/nu14102122 (PMC9146705; doi:10.3390/nu14102122)
Supplement: Supplementary file 1 [file nutrients-14-02122-s001.zip › nutrients-1720856-supplementary.pdf]

**Table S1. Inclusion and Exclusion Criteria of the Study Population.**

| INCLUSION CRITERIA | EXCLUSION CRITERIA |
|---|---|
| Maternal age 18-40 years old | Maternal alcohol/drugs abuse |
| Maternal Caucasian ethnicity | Maternal complete edentulism |
| Maternal pre-gestational | Maternal disease |
| $18.5 \leq \text{BMI} \leq 25$ or $\geq 30$ | (except for GDM in OB mothers) |
| Singleton spontaneous pregnancy | Maternal and fetal infections |
| Term delivery | Fetal malformations and chromosomal disorders |
